# Elevated TyG index is associated with increased risk of vitamin D deficiency among elderly patients with type 2 diabetes

**DOI:** 10.1038/s41598-024-67127-1

**Published:** 2024-07-12

**Authors:** Qunyan Xiang, Hu Xu, Youshou Liu, Wu Huang

**Affiliations:** 1 Department of Geriatrics, The Second Xiangya Hospital of Central South University, Changsha, 410011 Hunan China; 2 Institute of Aging and Age-Related Disease Research, Central South University, Changsha, 410011 Hunan China; 3 Department of Geriatrics, Peking University First Hospital, Beijing, 100034 China

**Keywords:** Triglyceride-glucose index, Vitamin D deficiency, Type 2 diabetes mellitus, Elderly people, Endocrine system and metabolic diseases, Nutrition disorders

## Abstract

Vitamin D deficiency (VDD) is associated with increased risk of type 2 diabetes mellitus (T2DM) and insulin resistance (IR). We aimed to investigate the association between the triglyceride-glucose (TyG) index that represents IR and VDD in elderly patients with T2DM. We enrolled 572 elderly participants with T2DM in this study. TyG index was calculated as ln [fasting triglyceride (TG, mg/dL) × fasting blood glucose (mg/dL)/2]. Serum 25-hydroxyvitamin D [25(OH)D] level below 50 nmol/L was defined as VDD. The association between the TyG index and the VDD risk was evaluated by multivariate logistic regression analysis. We observed a significant decreased 25(OH)D level with the increase of the TyG index in elderly diabetic patients, and a negative correlation between the TyG index and 25(OH)D level. The participants in the highest TyG quartile had a 2.40-fold higher risk of VDD than those in the lowest TyG index quartile [OR 2.40; 95% CI 1.47–3.92; *P* < 0.001]. The association persisted after adjustments for age, sex, smoking, obesity, insulin therapy, hypoglycemic agents’ medication, and some biochemical parameters. TyG index may be involved in the pathophysiology of VDD, which could be a predictor for VDD in elderly diabetic patients.

## Introduction

Vitamin D deficiency (VDD) has become a pressing issue globally, posing serious risks to health consequences. The global prevalence of VDD has been estimated to be 60–80%, and even higher in elderly population^1^. Vitamin D comes in two main forms, vitamin D2 (ergocalciferol) and vitamin D3 (cholecalciferol). Vitamin D3 from the skin and vitamin D2 from the diet are hydroxylated into 25-hydroxyvitamin D [25(OH)D] in the liver firstly and then into 1, 25-dihydroxyvitamin D [1,25(OH)_2_D] in the kidney^2^. 25(OH)D is the main storage form in the body and can reflect vitamin D status.

It has been known that VDD usually lead to abnormalities in calcium, phosphorous and bone homeostasis, which resulted in secondary hyperparathyroidism, decreased bone mineral density, and osteoporosis^2^. Recently, emerging evidence from observational and longitudinal cohort studies has demonstrated that VDD was associated with insulin resistance (IR) and type 2 diabetes mellitus (T2DM)^3–7^. However, the underlying mechanisms responsible for the relationship between vitamin D and T2DM have not fully understood. One hypothesis was that vitamin D could improve IR through enhancing glucose utilization^8,9^.

The triglyceride-glucose (TyG) index, which is determined as ln [fasting triglyceride (TG, mg/dL) × fasting blood glucose (mg/dL)/2], is a simple and credible marker for identifying IR^10^. Du et al. reported that TyG index associated most significantly with homeostatic model assessment of insulin resistance (HOMA-IR) than some indicators (such as visceral adiposity index, lipid accumulation product, and some traditional lipid ratios), and is better for identifying IR individuals early^11^. Besides, Huang et al. found a significant positive correlation between TyG index and HOMA-IR, and demonstrated that the TyG index is accurate and applicable for the identification of IR in middle-aged people of China^12^. In addition, a growing number of studies revealed that the TyG index was positively related to the risk of newly diagnosed T2DM and diabetic complications^13–16^.

Several studies have evaluated the relationships between the TyG index and vitamin D status in different populations. Mustafa et al. reported an inverse association between the TyG index and 25(OH)D level in adolescents^17^. Besides, Dhas et al. described that 25(OH)D level had a negative association with the TyG index in middle-aged patients with T2DM^3^. In addition, Jia et al. demonstrated that 25(OH)D level was negatively correlated to TyG index in male patients with T2DM, but not in female patients^18^. Moreover, we found that the TyG index could be an efficient predictor for the risk of VDD in elderly diabetic patients than younger adults^19^. Hence, the present study aimed to further explore the association between the TyG index and vitamin D status in elderly patients with T2DM.

## Method

### Study design and participants

**Figure 1:**
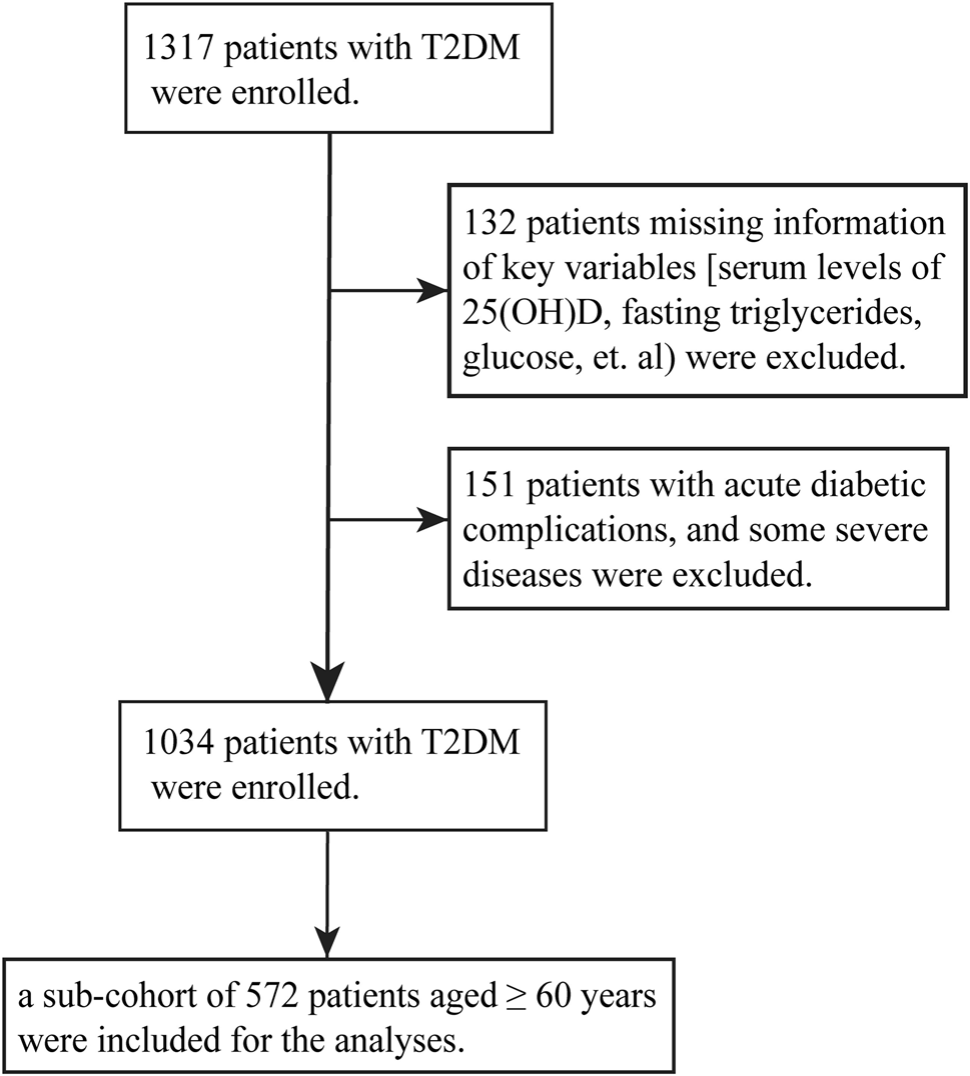
Flowchart of the participants enrolled in this study. The flowchart presented the procedure for inclusion and exclusion of the participants enrolled in the present study.

The participants were consecutively enrolled in the Second Xiangya Hospital of Central South University from September 2020 to October 2022, which has been described detailed previously^19^. Briefly, a total of 1034 participants diagnosed as T2DM were included in this cohort, and a sub-cohort of 572 elderly participants aged ≥ 60 years was selected in the present analyses. The detailed exclusion criteria were described previously^19^. VDD was defined as the serum level of 25(OH)D < 50 nmol/L. The flowchart presented the procedure for inclusion and exclusion of the study participants (Fig. 1).

The study was approved by the Ethics Committee of the Second Xiangya Hospital of Central South University ([2022]085), and carried out conformed to the Declaration of Helsinki. Informed consent was obtained from each participant.

### Data collection and biochemical detection

The demographic information, lifestyle, histories, previous medications, and some other basic clinical characteristics of the participants were collected at admission, and then experienced caregivers carried out the physical examination, including the body weight, height, and blood pressure. Blood samples were gained from all patients after an overnight fast, followed by biochemical detection. Fasting blood glucose (FBG), and blood lipids, including total cholesterol (TC), triglyceride (TG), low-density lipoprotein cholesterol (LDL-C), and high-density lipoprotein cholesterol (HDL-C) were measured on a HITACHI 7170A analyzer (Instrument Hitachi Ltd., Tokyo, Japan). Serum levels of C-peptide and glycated hemoglobin (HbA1c) were measured by ARCHITECT c8000 System (Abbott Laboratories, Irving, TX, USA) and Hemoglobin Testing System (VARIANT-11, Bio-Rad, Hercules, USA), respectively. Serum level of 25(OH)D was detected using chemiluminescence assay (Siemens ADVIA Centaur XP, Germany)^19^. According to suggestions from the research of China and abroad, a serum level of 25(OH)D < 50 nmol/L was considered as VDD^20,21^.

### Statistical analysis

The quantitative normally distributed data were expressed as the mean ± standard deviation (SD) and were compared using one-way ANOVA analysis among the four groups of the TyG index quartiles, while those with abnormally distributed data were presented as median and interquartile range, and were analyzed using Mann–Whitney U test between the two groups or Kruskal–Wallis test among the four groups. The qualitative data were described as numbers and percentages and were compared using Chi-square test for linear trend. The correlation between the TyG index and serum 25(OH)D level was performed by Spearman’s correlation analysis. The association of the TyG index with the risk of VDD was determined using multivariate logistic regression analyses: (1) model 1: unadjusted. Model 2: adjustments for age, sex, smoking, obesity, insulin therapy, and hypoglycemic agents’ medication. Model 3: adjustments for model 2 covariates plus body mass index (BMI), FBG, HbA1c, TC, LDL-C, and HDL-C. The predictive value of the TyG index for the risk of VDD was presented as odds ratio (OR) and 95% confidence interval (CI), and the lowest quartile of the TyG index was set as a reference. All statistical analyses were performed in SPSS software (version 25.0, Inc, Chicago, Illinois) or Graph Pad Prism software (version 7.0, Inc., LaJolla, CA). A two-tailed P value < 0.05 was considered as statistically significant difference.

### Ethical approval and consent to participate

The study was approved by the Ethics Committee of the Second Xiangya Hospital of Central South University ([2022]085), and carried out conformed to the Declaration of Helsinki. Each participate provided informed written consent.

## Results

### Clinical characteristics of the participants

A total of 572 elderly participants with T2DM were included in this study. Of the whole cohort, 311 were males (54.4%) and 261 were females (45.6%), and the age range was 60–94 years (average 70.05 ± 7.00 years). Participants were stratified into four groups based on the quartiles of the TyG index [Q1 (first quartile): 7.38 ≤ TyG ≤ 8.51, Q2 (second quartile): 8.52 ≤ TyG ≤ 8.95, Q3 (third quartile): 8.96 ≤ TyG ≤ 9.42, Q4 (fourth quartile): 9.43 ≤ TyG ≤ 11.34)]. Participants in the higher quartiles of the TyG index showed increased levels of BMI, TC, TG, LDL-C, FBG, HbA1c, and C-peptide, while decreased 25(OH)D and HDL-C levels, when compared with those in the lowest TyG index quartile (all *P* for trend < 0.05). In addition, sex, smoking, obesity, and insulin therapy were significantly different among groups (all *P* for trend < 0.05). No significant differences were found in terms of age, hypertension, SBP, DBP, the usage of calcium channel blockers (CCB), angiotensin-converting enzyme inhibitors (ACEI), β blockers, oral hypoglycemia agents, and statin (all *P* for trend > 0.05) (Table 1).

**Table 1:** Clinical characteristics of the participants based on the quartiles of the TyG index.

	Quartiles of the TyG index				*P* for trend
	Q1 (7.38–8.51)	Q2 (8.52–8.95)	Q3 (8.96–9.42)	Q4 (9.43–11.34)	
Age, years	70.17 ± 7.16	71.24 ± 7.36	69.02 ± 6.53	69.76 ± 6.81	0.056
Sex, n (%)					0.017
Males	94 (65.73)	73 (51.05)	70 (48.95)	74 (51.75)	
Females	49 (34.27)	70 (48.95)	73 (51.05)	69 (48.25)	
Smoking, n (%)	55 (38.5)	33 (23.1)	43 (30.1)	40 (28.0)	0.042
Obesity, n (%)	13 (9.1)	9 (6.3)	17 (12.0)	24 (16.8)	0.026
Hypertension, n (%)	100 (69.9)	109 (76.2)	100 (69.9)	107 (74.8)	0.507
Antihypertensive drugs					
CCB, n (%)	81 (56.6)	89 (62.2)	92 (64.3)	90 (62.9)	0.558
ACEI, n (%)	30 (21.0)	29 (20.3)	34 (23.8)	32 (22.4)	0.896
β blockers, n (%)	72 (50.3)	66 (46.2)	70 (49.0)	71 (49.7)	0.901
Insulin therapy, n (%)	79 (55.2)	69 (48.3)	79 (55.2)	97 (67.8)	0.009
Oral hypoglycemia agents, n (%)	117 (81.8)	129 (90.2)	129 (90.2)	122 (85.3)	0.098
Statin therapy, n (%)	105 (73.4)	103 (72.0)	112 (78.3)	115 (80.4)	0.294
BMI, kg/m^2^	23.63 ± 3.84	23.82 ± 3.16	23.92 ± 3.40	25.06 ± 3.09	0.002
SBP, mmHg	139.56 ± 21.20	137.93 ± 19.93	138.46 ± 21.20	141.13 ± 21.95	0.587
DBP, mmHg	77.08 ± 10.64	77.27 ± 10.36	78.17 ± 10.42	79.90 ± 14.34	0.151
TC, mmol/L	3.79 ± 0.92	4.17 ± 1.03	4.34 ± 1.26	4.71 ± 1.29	< 0.001
TG, mmol/L	0.80 (0.65, 0.96)	1.20 (1.03, 1.43)	1.67 (1.32, 1.98)	2.52 (2.10, 3.19)	< 0.001
LDL-C, mmol/L	2.18 ± 0.84	2.60 ± 0.93	2.76 ± 1.08	3.05 ± 1.17	< 0.001
HDL-C, mmol/L	1.25 ± 0.34	1.15 ± 0.29	1.06 ± 0.29	0.98 ± 0.22	< 0.001
FBG, mmol/L	5.79 (4.88–6.60)	6.30 (5.50–7.31)	7.41 (6.35–8.68)	8.59 (7.22–10.59)	< 0.001
HbA1c, %	7.47 (6.39–8.57)	8.25 (6.86–9.36)	9.03 (7.72–10.25)	8.85 (7.39–10.19)	< 0.001
C-peptide, pmol/L	293.95 (182.35–526.28)	345.20 (210.05–480.60)	385.40 (229.35–578.40)	419.25 (278.88–665.83)	< 0.001
25(OH)D, nmol/L	48.00 (39.00–63.00)	47.50 (34.75–65.25)	46.00 (37.00–59.00)	44.00 (36.00–56.25)	0.003
TyG index	8.17 ± 0.28	8.74 ± 0.13	9.17 ± 0.14	9.84 ± 0.40	< 0.001

Data were given as mean ± standard deviation (SD) or median (25–75% interquartile range) for normally and abnormally distributed data, respectively. The qualitative data were presented as n (%).

*TyG index* triglyceride-glucose index, *CCB* calcium channel blockers, *ACEI* angiotensin-converting enzyme inhibitors, *BMI* body mass index, *SBP* systolic blood pressure, *DBP* diastolic blood pressure, *TC* total cholesterol, *TG* triglyceride, *LDL-C* low-density lipoprotein cholesterol, *HDL-C* high-density lipoprotein cholesterol, *FBG* fasting blood glucose, *HbA1c* glycated haemoglobin, *25(OH)D* 25-hydroxyvitamin D.

### Correlation between the TyG index and 25(OH)D

**Figure 2:**
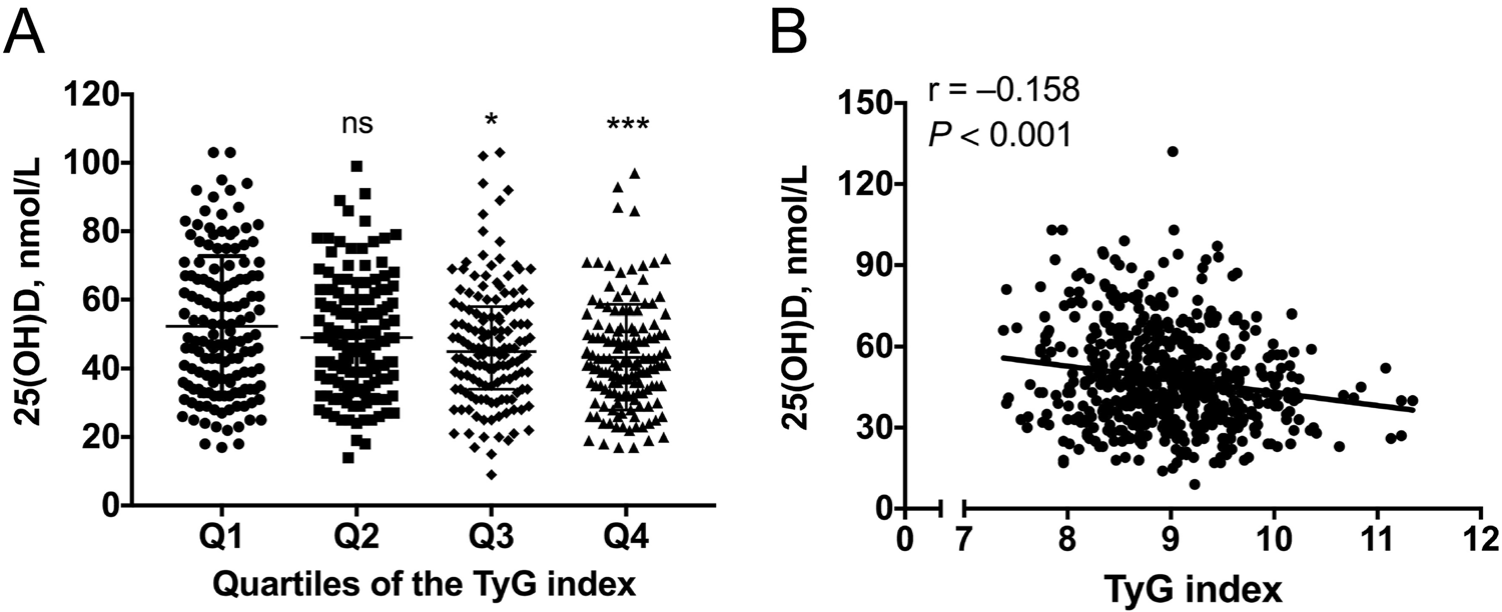
Association between the TyG index and serum 25(OH)D level. (**A**) Comparisons of 25(OH)D level among the quartiles of the TyG index. (**B**) Spearman’s correlation between the TyG index and 25(OH)D level. *P < 0.05, ***P < 0.001, compared with the Q1 quartile of the TyG index. *ns* no significant, *TyG index* triglyceride-glucose index, *25(OH)D* 25-hydroxyvitamin D.

Compared with the participants in the lowest TyG index quartile, those in the higher quartiles of the TyG index had an increased trend of serum 25(OH)D level, with statistically significance were observed among Q3 and Q4 groups (all *P* < 0.05) (Table 1, Fig. 2A). Besides, a negative correlation was showed between the TyG index and serum 25(OH)D level (Spearman’s rho = − 0.158, *P* < 0.001, Fig. 2B).

### Association between the TyG index and the risk of VDD

**Figure 3:**
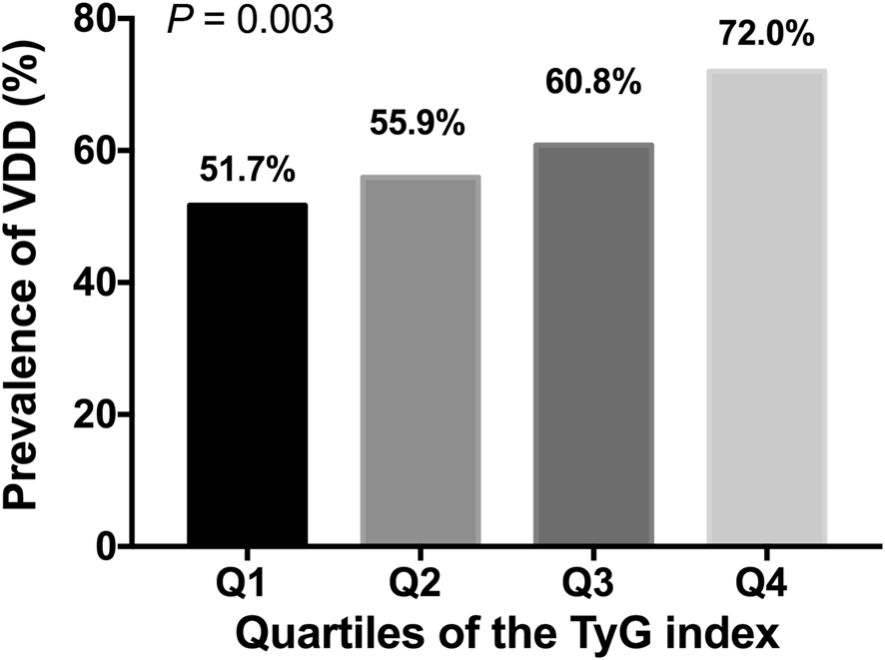
VDD prevalence among the TyG index quartiles. *VDD* vitamin D deficiency, *TyG index* triglyceride-glucose index.

To explore the relationship between the TyG index and VDD, the percentages of VDD prevalence among the TyG index quartiles were compared. As shown in Fig. 2, an increased VDD prevalence was observed in line with the increase of the TyG index quartiles (51.7%, 55.9%, 60.8%, 72.0% in the Q1, Q2, Q3 and Q4 quartiles, *P* for trend = 0.003, Fig. 3).

To further explore the predictive value of the TyG index for the risk of VDD, multivariate logistic regression analyses were used. It was showed that when taking the lowest quartile of the TyG index as a reference, the risks of VDD were increased in another three groups, with the a 2.40-fold higher risk of VDD in the highest TyG quartile [OR 2.40; 95% CI 1.47–3.92; *P* < 0.001]. An increased trend of VDD prevalence in line with the increase of the TyG index quartiles was still observed after adjustments for clinical and laboratory parameters. Participants in the highest TyG index quartile showed statistically increased risk of VDD when compared with those in the lowest TyG index quartile after adjustments for age, sex, smoking, obesity, insulin therapy, hypoglycemic agents’ medication [OR 2.44; 95% CI 1.45–4.10; *P* < 0.001]. Additionally, a slightly higher risk was observed in the highest TyG index quartile after further adjustments for BMI, FBG, HbA1c, TC, LDL-C, and HDL-C [OR 2.89; 95% CI 1.33–6.24; *P* < 0.001] (Table 2).

**Table 2:** Association of the TyG index with VDD prevalence.

TyG index quartiles	Model 1		Model 2		Model 3	
	OR (95% CI)	*P*	OR (95% CI)	*P*	OR (95% CI)	*P*
Q1	1		1		1	
Q2	1.18 (0.74–1.19)	0.477	1.31 (0.80–2.13)	0.285	1.13 (0.65–1.94)	0.671
Q3	1.45 (0.91–2.32)	0.122	1.53 (0.94–2.51)	0.090	1.54 (0.84–2.84)	0.167
Q4	2.40 (1.47–3.92)	< 0.001	2.44 (1.45–4.10)	0.001	2.89 (1.33–6.24)	0.007

Model 1: unadjusted. Model 2: adjustments for age, sex, smoking, obesity, insulin therapy, and hypoglycemic agents’ medication. Model 3: adjustments for model 2 covariates plus BMI, FBG, HbA1c, TC, LDL-C, and HDL-C.

*TyG index* triglyceride-glucose index, *VDD* vitamin D deficiency, *BMI* body mass index, *FBG* fasting blood glucose, *HbA1c* glycated hemoglobin, *TC* total cholesterol, *LDL-C* low-density lipoprotein cholesterol, *HDL-C* high-density lipoprotein cholesterol.

## Discussion

In this study, we observed a significant decreased level of 25(OH)D with the increase of the TyG index in elderly patients with T2DM, and a negative correlation between the TyG index and 25(OH)D level. Further exploration showed that elevated TyG index increased the risk of VDD in elderly diabetic patients. These results suggested that TyG index was involved in the development of VDD and could be a predictor for VDD.

Exposure to sunlight is the major source of vitamin D for most people. However, a variety of factors influence the amount of sunlight hitting to the skin and the cutaneous of production of vitamin D, such as age, skin color, sunscreen use, clothing, season, lifestyle, altitude, and latitude^22^. The generally accepted assessment of vitamin D status is measuring circulating 25(OH)D level, but the definition of VDD varied. It has been suggested that the values of 25(OH)D below 50 nmol/L (20 ng/mL) is considered as VDD in China^21,23,24^. The prevalence of VDD varies in different regions. It has been estimated that almost 60%–80% of the people had VDD or vitamin D insufficiency^1,2^. In China, the prevalence of VDD was 70–90% in northern cities, while almost 50% in southern cities^21,23–25^. The prevalence of VDD was higher in individuals with T2DM than those without T2DM^7^. Besides, the prevalence of VDD was higher in elderly population due to less sun exposure and reduced dietary vitamin D intake^26^. Elderly individuals with VDD are at high risk for falls and fractures^27^. In this study, the prevalence of VDD was 60.1% in elderly people with T2DM, which was close to that in our previous study^19^. This may be related to the relatively higher use of vitamin D supplements in elderly.

The main physiologic functions of vitamin D is to maintain the balance of bone metabolism through promoting the absorption of calcium and phosphorus and inhibiting the release of parathyroid hormone^2^. Recently, with the identification of vitamin D receptor in multiple tissues and cells, the biological functions of vitamin D has been recognized, such as stimulating insulin production and enhancing glucose utilization^8,9^. Emerging evidence from observational and longitudinal cohort studies demonstrated that vitamin D could alleviate IR and decrease the risk of T2DM^3,6,7^. However, conclusion from randomized controlled trials were controversial^28^. Results from some studies supported the benefits of vitamin D supplementation on pancreatic beta cell function^29^, while those from other studies suggested that vitamin D supplementation did not affect insulin secretion and T2DM development^30,31^. Cojic et al.^32^ reported that oral daily doses of vitamin D supplementation improved HbA1c levels and decreased the oxidation protein products levels when given higher doses of vitamin D. They also found that vitamin D supplementation improved the endothelial dysfunction in diabetic patients through reducing the production of reactive oxygen species and inflammation^33^. In this observational study, we found a decreased 25(OH)D level in diabetic patients with high TyG index than those with lower TyG index. This suggested that vitamin D status was negatively associated with IR, which was consistent with the findings of most observational studies.

Although numerous studies reported the role of vitamin D on the pathogenesis of IR or T2DM, few studies focused on the role of IR on VDD development. Adipose tissue is the main storage site for circulating 25(OH)D. It has been known that obese individuals were more likely to have lower 25(OH)D level in circulation due to its deposition in adipose tissue^34^. The mechanisms may be related to impaired metabolism^35^, decreased sequestration^36^, and declined release of vitamin D^37^ in obese IR state. Our previous and the present studies found that high TyG index is associated with increased risk of VDD^19^. Elderly diabetic individuals in the highest quartile of the TyG index showed a 2.40-fold higher risk of VDD when compared to those in the lowest TyG index quartile. Based on these evidences, it was speculated that high TyG index representing IR may be involved in the pathogenesis of VDD in T2DM. But this needs to be verified in large-scale cohort studies and randomized controlled trials, and the underlying mechanisms remain to be further clarified.

IR is a main feature as well as a key pathophysiological mechanism for T2DM. Euglycemic–hyperinsulinemic clamp test is the gold standard for the evaluation of IR, but it is relatively invasive and expensive, which is limited to clinical application^38^. HOMA-IR has been proposed to replace the euglycemic-hyperinsulinemic clamp test for assessing IR^39^. However, the strict requirements of HOMA-IR, such as no use drugs that affect the metabolism of intrinsic insulin, have also limited its application. TyG index has been confirmed to be a simple and accurate marker for the assessment of IR. Emerging evidence has demonstrated that TyG index associated significantly with HOMA-IR, and is an appropriate indicator for identifying IR^11,12^. Besides, it has been revealed that the TyG index was associated positively to the risk of T2DM and diabetic complications^13–16^. Hence, we used TyG index as a marker for IR in the present study.

The major findings of this study highlighted the association of elevated TyG index with increased risk of VDD in elderly individuals with T2DM from the central part of China. These findings provided a reference for future explorations of the association between IR and VDD. Still, there were several limitations in our study. Firstly, it was a retrospective study without investigation on any cause and effect relationship. Secondly, the study was carried out in a central part of China and the conclusions may not represent the situation elsewhere. Thirdly, the sample size was relatively small, which needs to be further studied in large cohort and prospective studies.

In summary, this study found that the TyG index was negatively associated with serum 25(OH)D level in elderly individuals with T2DM. Besides, with the increase of the TyG index, the prevalence of VDD showed an increasing trend. These results suggested that TyG index representing IR may be involved in the pathogenesis of VDD. Future studies are required to focus on the mechanisms of IR on VDD.

## Data Availability

Data will be made available from the corresponding author on request.

## Abbreviations

VDD: Vitamin D deficiency
25(OH)D: 25-Hydroxyvitamin D
1,25(OH)_2_D: 1,25-Dihydroxyvitamin D
IR: Insulin resistance
T2DM: Type 2 diabetes mellitus
TyG: Triglyceride-glucose
TG: Triglyceride
HOMA-IR: Homeostatic model assessment of insulin resistance
BMI: Body mass index
FBG: Fasting blood glucose
LDL-C: Low-density lipoprotein cholesterol
HDL-C: High-density lipoprotein cholesterol
HbA1c: Glycated hemoglobin
SD: Standard deviation
OR: Odds ratio
CI: Confidence interval
CCB: Calcium channel blockers
ACEI: Angiotensin-converting enzyme inhibitors
